# *ProSPyX*: software for post-processing images of X-ray ptychography with spectral capabilities

**DOI:** 10.1107/S160057752400016X

**Published:** 2024-02-09

**Authors:** Redhouane Boudjehem, Anico Kulow, Javier Pérez, Eric Gautier, Samy Ould-chikh, Sébastien Pairis, Jean-Louis Hazemann, Julio César da Silva

**Affiliations:** a Université Grenoble Alpes, CNRS, Grenoble INP, Institut Néel, 25 Avenue des Martyrs, BP 166, 38042 Grenoble, France; b Synchrotron SOLEIL, Saint-Aubin, France; c SPINTEC, Université Grenoble Alpes, CEA, CNRS, 17 rue des Martyrs, 38054 Grenoble, France; d King Abdullah University of Science and Technology, KAUST Catalysis Center, Advanced Functional Materials, Thuwal 23955, Saudi Arabia; Paul Scherrer Institut, Switzerland

**Keywords:** *ProSPyX*, X-ray spectral ptychography, graphical user interface, *PyQt5*, *Toupy*

## Abstract

X-ray ptychography is a coherent diffraction imaging technique based on acquiring multiple diffraction patterns obtained through the illumination of the sample at different partially overlapping probe positions. The diffraction patterns collected are used to retrieve the complex transmittivity function of the sample and the probe using a phase retrieval algorithm. Absorption or phase contrast images of the sample as well as the real and imaginary parts of the probe function can be obtained. Furthermore, X-ray ptychography can also provide spectral information of the sample from absorption or phase shift images by capturing multiple ptychographic projections at varying energies around the resonant energy of the element of interest. However, post-processing of the images is required to extract the spectra. To facilitate this, *ProSPyX*, a Python package that offers the analysis tools and a graphical user interface required to process spectral ptychography datasets, is presented. Using the *PyQt5* Python open-source module for development and design, the software facilitates extraction of absorption and phase spectral information from spectral ptychographic datasets. It also saves the spectra in file formats compatible with other X-ray absorption spectroscopy data analysis software tools, streamlining integration into existing spectroscopic data analysis pipelines. To illustrate its capabilities, *ProSPyX* was applied to process the spectral ptychography dataset recently acquired on a nickel wire at the SWING beamline of the SOLEIL synchrotron.

## Abstract 2

*ProSPyX*is a Python intuitive graphical user interface that enables extraction of absorption and phase spectral information from spectral ptychographic datasets.

## 1. Introduction

Far-field X-ray ptychography requires a sample to be scanned across a coherent beam. By 2D scanning the sample, a series of overlapping diffraction patterns are recorded by a detector placed in the far field (Rodenburg *et al.*, 2007 ▸; Thibault *et al.*, 2008 ▸; da Silva & Menzel, 2015 ▸). The detected intensity for each diffraction pattern corresponds to the modulus squared of the Fourier transform of the exit wave past the sample and the phase information is lost. The exit wave can be mathematically represented by the product of the sample transmittivity function and the probe function, which are both complex-valued functions. The redundancy introduced by the overlapping scan positions allows the retrieval of the lost phase, thereby reconstructing the complex-valued functions of the transmittivity of the sample and of the probe. Such a phase-retrieval strategy relies on iterative algorithms (Fienup, 1982 ▸; Maiden & Rodenburg, 2009 ▸; Thibault *et al.*, 2009 ▸). Two examples of ptychographic phase retrieval software are the open-source Python-based package *Ptypy* (Enders & Thibault, 2016 ▸) and the *PtychoShelves* software (Wakonig *et al.*, 2020 ▸), which is open-source but requires a valid commercial license of *Matlab*.

By combining X-ray ptychography with computed tomography (Dierolf *et al.*, 2010 ▸; Guizar-Sicairos *et al.*, 2011 ▸) one can obtain a quantitative 3D reconstruction of the microstructure of the sample. Ptychographic X-ray computed tomography (PXCT) is achieved by performing multiple 2D ptychography projection acquisitions at different angles between 0 and 180° or between 0 and 360°. Afterwards, the tomographic projections are processed, including the correction of linear phase ramp removal (Guizar-Sicairos *et al.*, 2011 ▸), 2D phase unwrapping (Goldstein *et al.*, 1988 ▸; Flynn, 1997 ▸) and the alignment of the projections. Once all this is done, the absorption and phase-contrast tomograms are reconstructed using standard tomographic reconstruction algorithms (Natterer, 2001 ▸; Herman, 2009 ▸).

X-ray spectral ptychography (Beckers *et al.*, 2011 ▸; Maiden *et al.*, 2013 ▸; Shapiro *et al.*, 2014 ▸) is a variant of X-ray ptychography that adds spectral information to the reconstructed images. This allows for the determination of not only the spatial structure of the sample but also its chemical composition. X-ray spectral ptychography was applied to emerging questions in energy materials such as catalysts, batteries and magnetic materials. In the catalysis field, a study conducted by Hirose *et al.* (2018 ▸) examined platinum-supported cerium–zirconium oxide three-way catalyst particles using Ce *L*-edge chemical mapping with a phase retrieval algorithm constrained by the Kramers–Kronig relation between absorption and phase spectra. In the batteries sector, X-ray spectral ptychography was used by Yu *et al.* (2015 ▸) to observe the relationship between the mechanical and the chemical stability of lithium-ion batteries and battery cycling. In the magnetism domain, the magnetic nanostructure of materials can be examined by spectral ptychography using X-ray magnetic circular dichroism, which is sensitive to the magnetic strength and alignment of ferromagnetic phases. Gräfe *et al.* (2020 ▸) used Fe *L*_3_-edge X-ray magnetic circular dichroism contrast spectral ptychography to examine antidot lattices, where the improved spatial resolution of these ptychography experiments enabled imaging of magnetic domains in a sample with topological solid variation.

**Figure 1:**
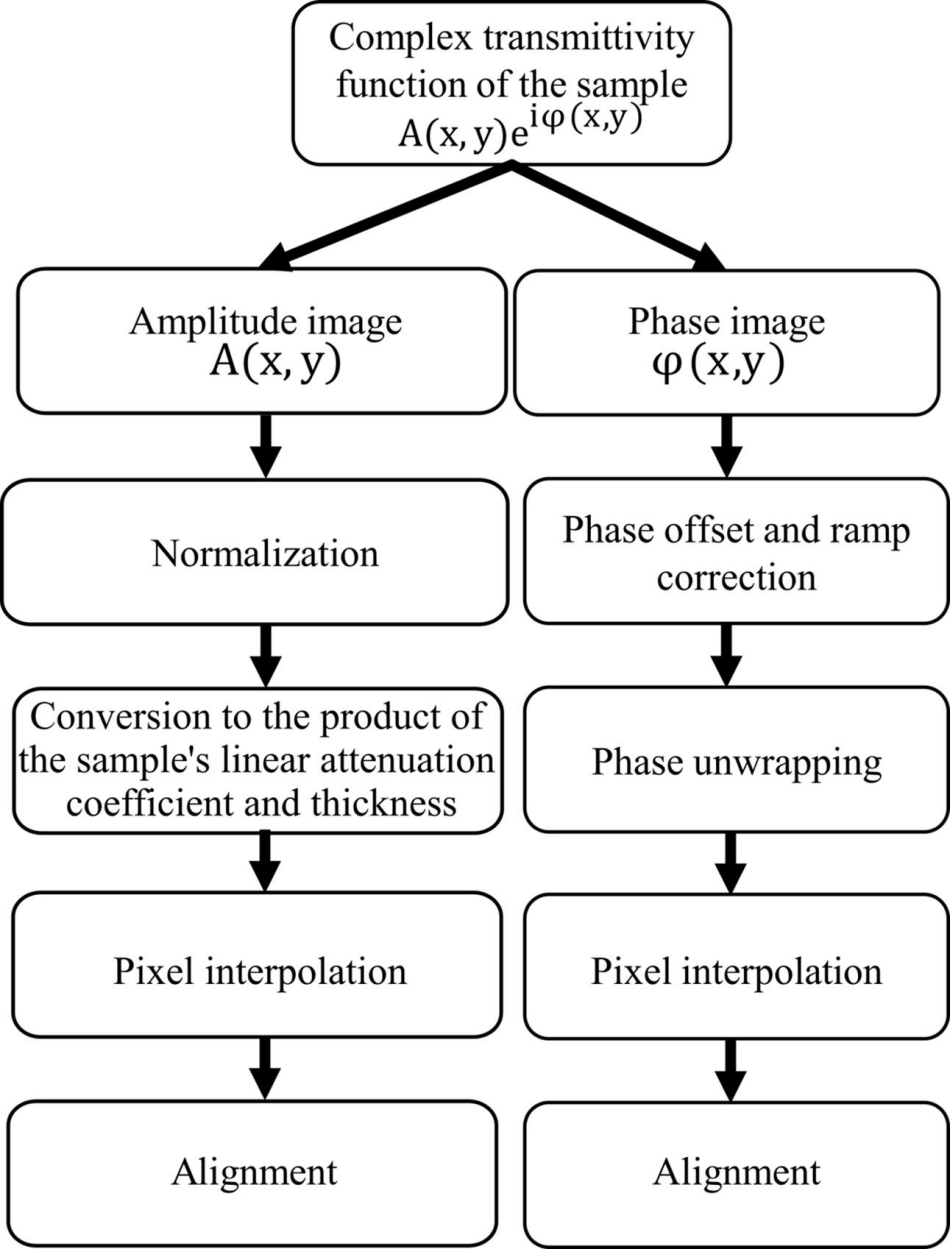
Post-processing pipeline of the spectral ptychographic 2D images after phase retrieval. Every phase contrast image must undergo phase offset and ramp correction, phase unwrapping, pixel interpolation, and alignment. Every amplitude image has to undergo normalization, conversion to the product of the sample linear attenuation coefficient and thickness, pixel interpolation, and alignment.

X-ray spectral ptychography is performed by collecting diffraction patterns over a range of X-ray energies around an absorption edge of an element of interest. The complex transmittivity function of the sample is then reconstructed for each energy. As a result, a set of phase shift and absorption contrast images is obtained. To allow the proper spectral analysis, each set goes through a defined post-processing pipeline (see Fig. 1 ▸), including constant phase offset and linear phase ramp removal, phase unwrapping, pixel interpolation, and alignment. For the absorption set, the images need to be normalized and converted to the product of the linear attenuation coefficient and thickness of the sample, and pixel interpolation and alignment also need to be done.

Numerous Python packages provide the tools to build the post-processing pipeline. *Toupy* is an open-source Python package that focuses on data post-processing of 2D ptychography images and tomography reconstruction (Da Silva, 2019 ▸). However, *Toupy* does not provide all the required tools for X-ray spectral ptychography. In addition, developing software for post-processing X-ray spectral ptychography is important for several reasons: (i) software can automate the post-processing process, reducing the time and effort required to produce high-resolution images; (ii) it can perform complex calculations and data analysis more accurately and consistently than manual methods, leading to more accurate and reliable results; and finally, (iii) the software can be shared and used by multiple researchers, facilitating the sharing of data and results across institutions. To the best of our knowledge, no software offering a complete post-processing pipeline has been proposed. Therefore, we present *ProSPyX*, an open-source graphical user interface (GUI) written in Python and designed with the *PyQt5* Python open-source module. Unlike the *Toupy* package, *ProSPyX* allows the post-processing of X-ray spectral ptychography datasets. It also enables the extraction of spectra from the absorption and phase-contrast images to inspect the presence of the resonant chemical species. The discussion in this article is organized as follows: after a description of the software structure, we discuss how to properly import and process the spectral phase shift and absorption images. Then, we show how absorption and phase spectral information are extracted from the images. The capabilities of *ProSPyX* are illustrated by the processing of a recently acquired X-ray spectral ptychographic dataset on a nickel wire at the SWING beamline of the SOLEIL synchrotron.

## 2. Software structure

*ProSPyX* relies on Python with an object-oriented programming paradigm. It contains one class called ‘MainWindow’. Inside this class, the setup of the GUI is built, and the instance methods for post-processing are written. The software is suitable for ordinary computers running under any operating system as long as Python and its dependencies are installed. At the time of this work, it had been tested using Python v3.9. The source code and the latest developments of *ProSPyX* are available on GitHub (https://github.com/RedhouaneBJM/ProSPyX).

The software is constructed using the *QtCore*, *QtGui* and *Qtwidgets* modules, each serving a specific purpose. The *QtCore* module provides core classes that facilitate the *Qt* event loop, signaling, slot mechanisms and application settings. Meanwhile, the *QtGui* module contains classes that enable integration with the windowing system, handle events, basic image handling, fonts and text. These classes are used internally by the *Qt* user interface technology but can also be used directly, such as when developing applications that use the low-level OpenGL ES graphics API. Finally, the *Qtwidgets* module provides a collection of user interface components to build traditional desktop-style user interfaces. The instance methods of the ‘MainWindow’ are controlled interactively by the user interface elements of the *Qt* widget module.

**Figure 2:**
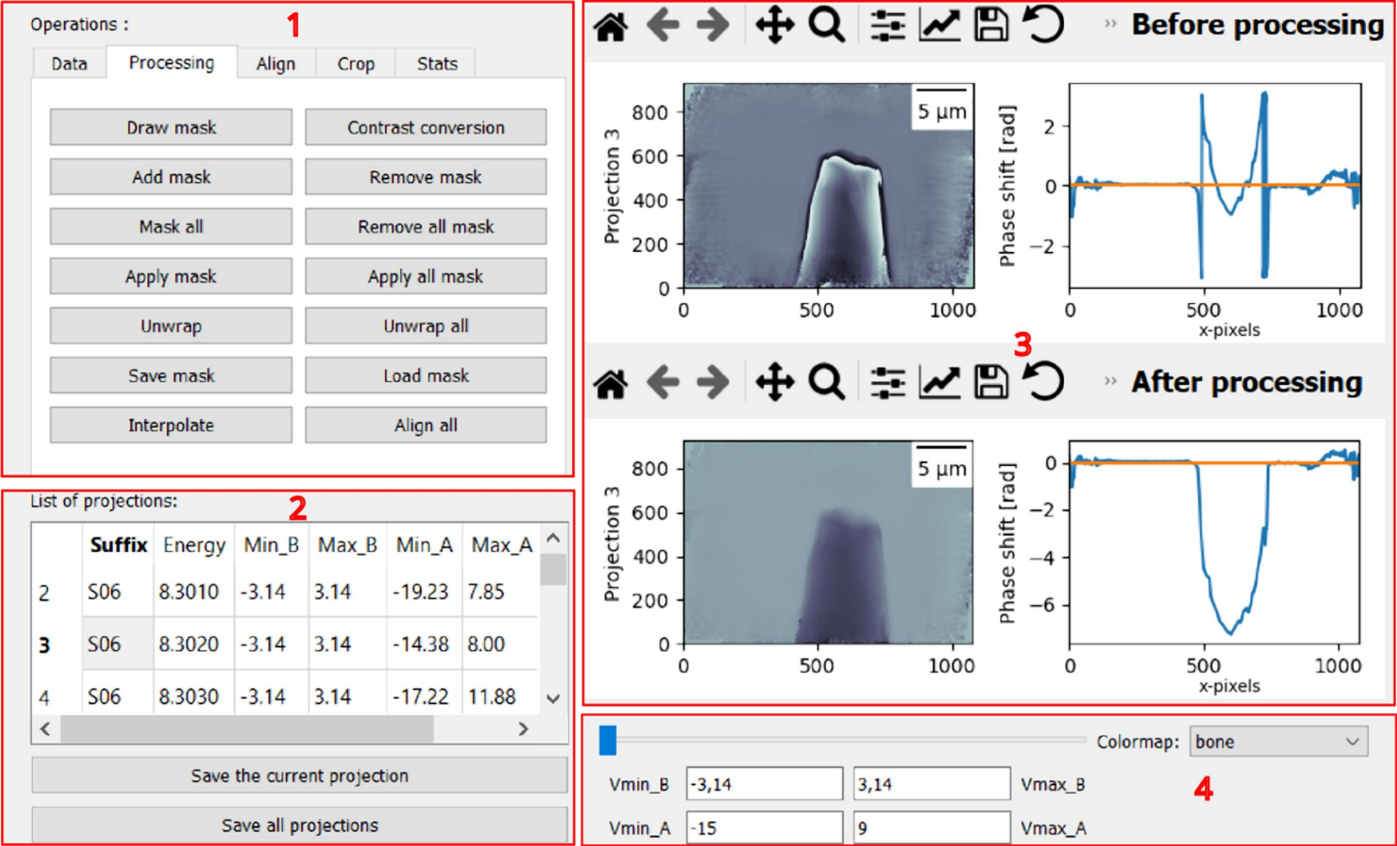
*ProSPyX* software main window. The software is divided into four areas: (1) reading and processing area, (2) listing and saving data area, (3) displaying area before and after processing, and (4) editing display parameter area.

**Figure 3:**
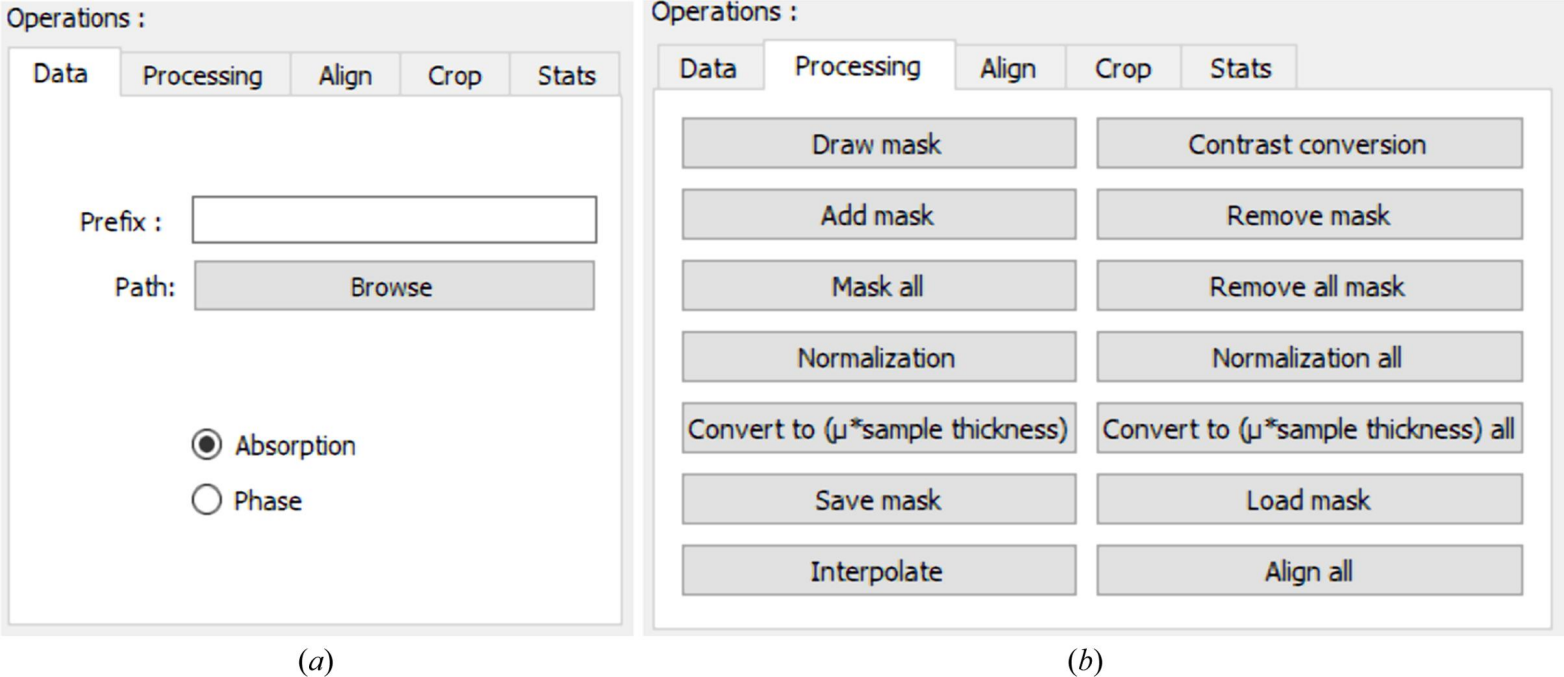
Data reading and contrast change tab in the *ProSPyX* software. (*a*) The user must first provide the prefix and validate it by pressing the enter key and then pressing ‘Browse’ to define the path where the data are stored, considering that all the files have the same prefix in their names. The ‘Phase’ and ‘Absorption’ radio buttons allow the user to switch between the phase and absorption contrasts. (*b*) Push buttons for processing absorption contrast images that appear after switching to this contrast.

The GUI window is divided into four areas where, within the first area, there are five tabs (see Fig. 2 ▸). Inside the first area, called ‘Operations’, the first tab ‘Data’ is for reading data [see Fig. 3 ▸(*a*)], which allows the user to read the stack of files considering that all of them have the same prefix in their names. Moreover, this tab provides the option to switch between the phase and absorption contrasts.

**Figure 4:**
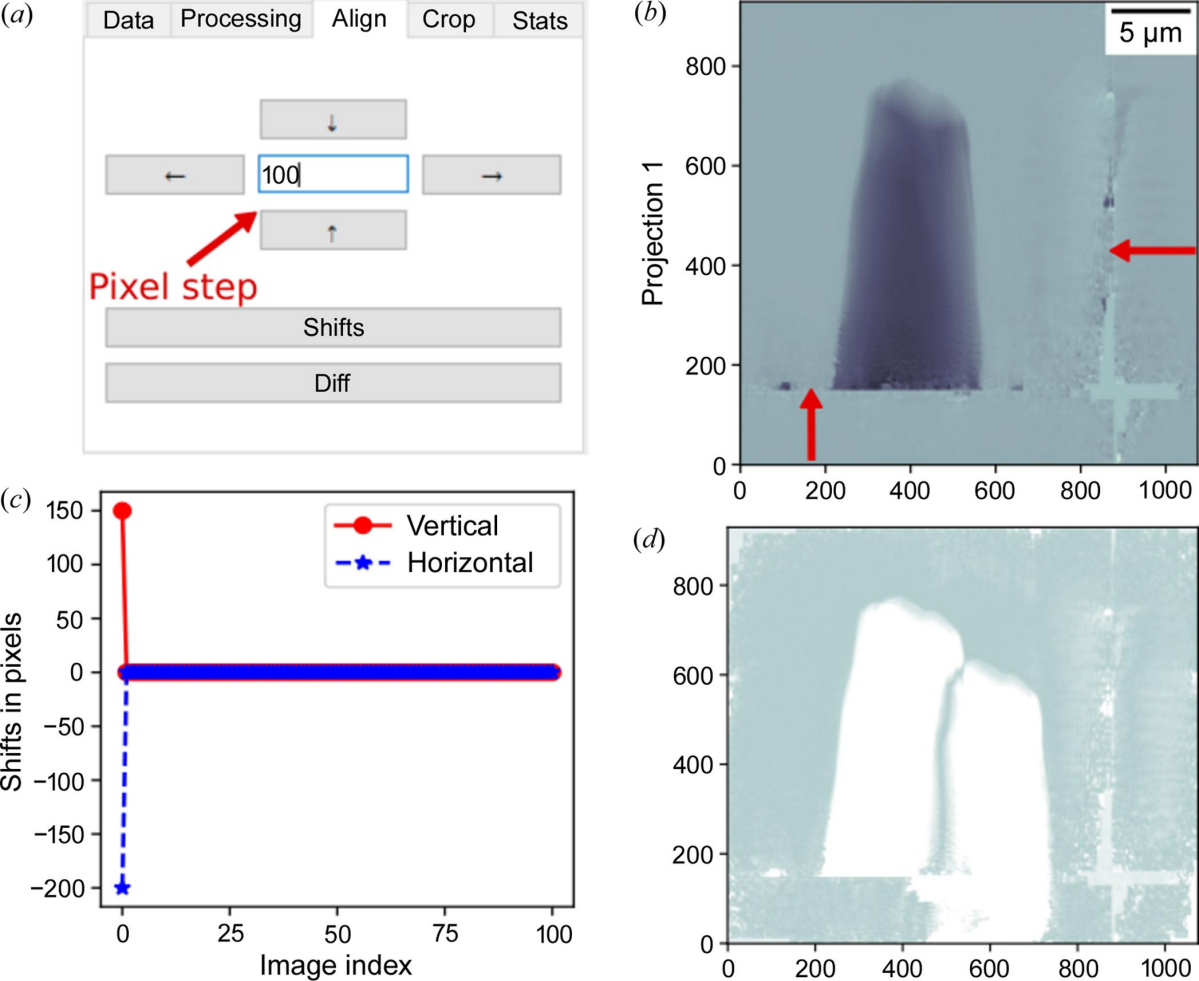
Manual alignment with the *ProSPyX* software. (*a*) Buttons with the arrows indicate the direction of the shifts with a shifting step provided by the user. (*b*) Selected moving image. (*c*) ‘Shifts’ button plots the horizontal and vertical introduced shifts. (*d*) ‘Diff’ button displays the difference between the reference and selected moving images.

The second tab is for applying post-processing algorithms to the phase or absorption contrast images after phase retrieval. The third tab is dedicated to manual alignment (see Fig. 4 ▸) in cases where the automatic alignment in the second tab does not provide accurate results. This is done by introducing unlimited horizontal or vertical shifts using the four push buttons with arrows that indicate shift directions with a pixel-wide step defined by the user. It is also possible to plot the introduced shifts between the reference and moving images [see Fig. 4 ▸(*c*)]. As in most registration algorithms, the reference image is used as a template for the registration, while the moving one is the image that needs to be aligned to match the reference. For the sake of simplicity here, the reference image refers to the image acquired with the highest energy.

**Figure 5:**
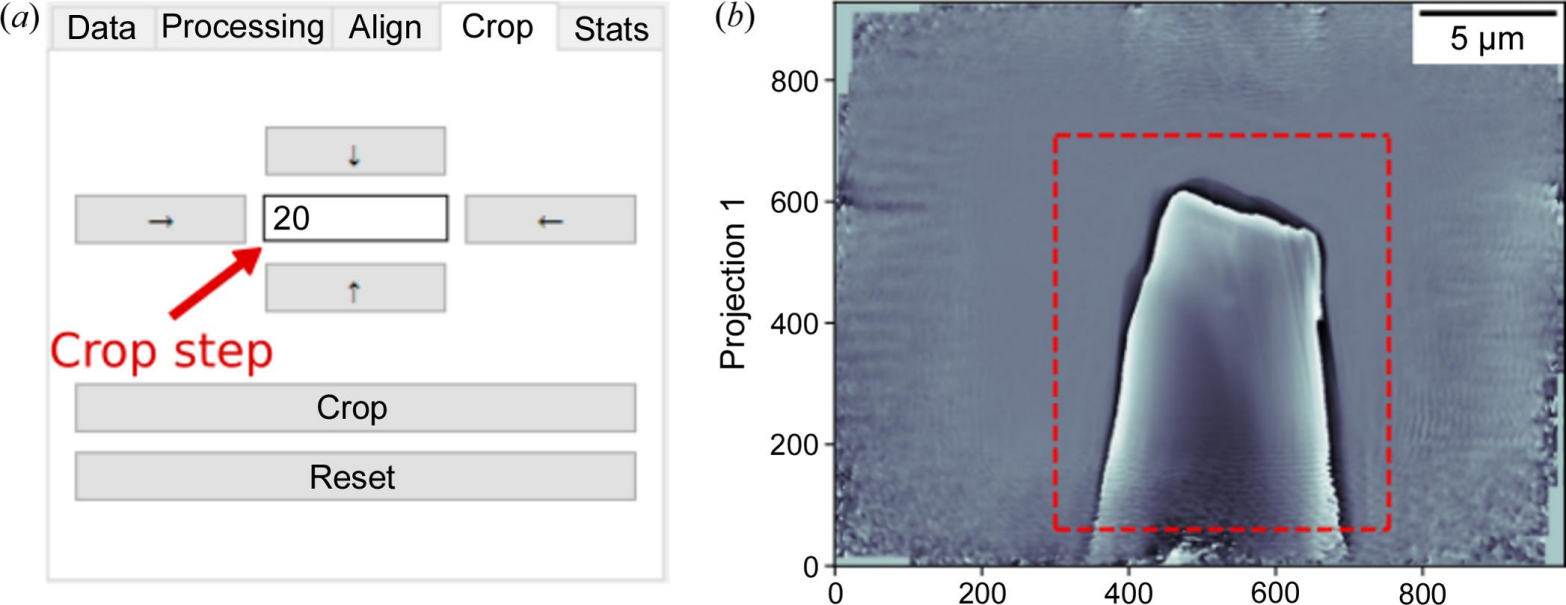
Cropping with the *ProSPyX* software. (*a*) Buttons with arrows define the region to isolate using the ‘Crop’ button. ‘Reset’ undoes the defined region. (*b*) The dashed-red rectangle defined using the cropping tab delimits the region to be isolated.

The fourth tab (see Fig. 5 ▸) allows cropping in a rectangular or square form using the four push buttons to select the region to keep once the pixel step is defined.

**Figure 6:**
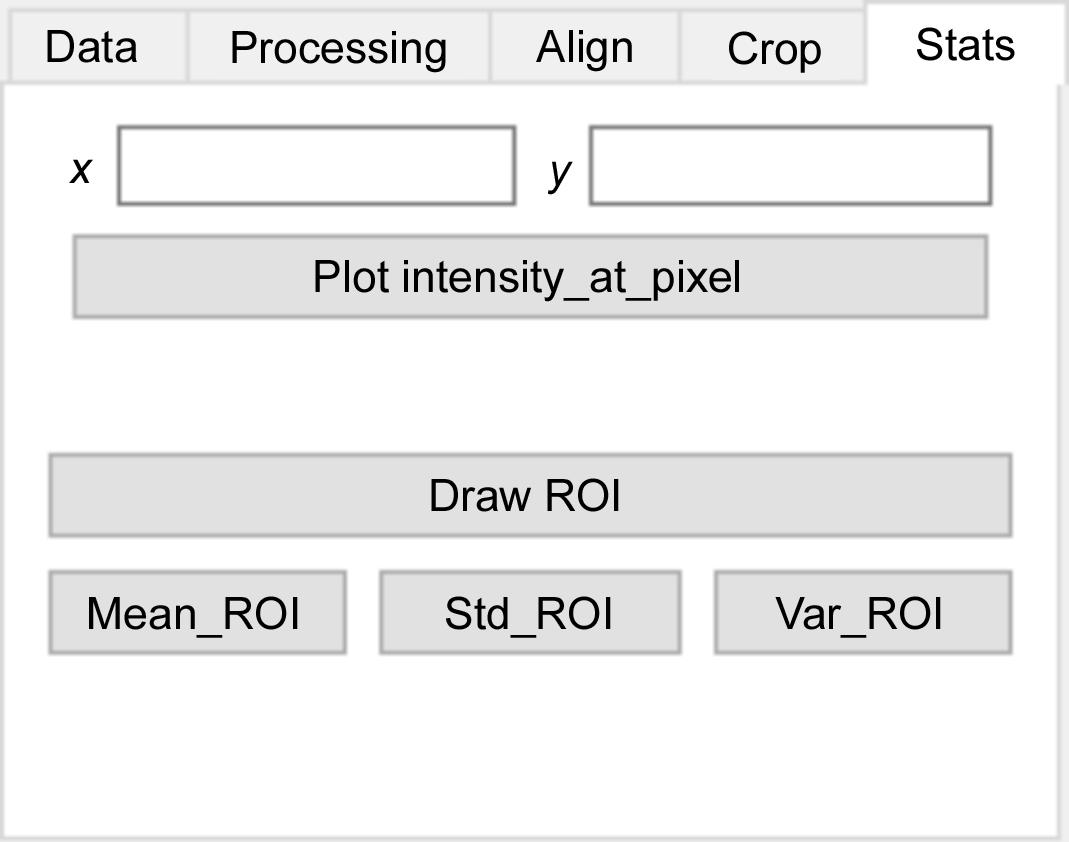
Stats tab of *ProSPyX* software. The user can plot the intensity variation of one pixel at coordinates (*x*, *y*); or the mean, variance or standard deviation of a set of pixels within a defined ROI for a given energy.

The last tab (see Fig. 6 ▸) permits either the plot intensity variation of one pixel at coordinates (*x*, *y*) or the mean, variance or standard deviation of a set of pixels within a user-defined region of interest (ROI) for a given energy.

The second area of the GUI lists the images that have been read with additional information, such as the energy of the acquisition ‘Min_B’, ‘Max_B’, ‘Min_A’ and ‘Max_A’, which are the minimum and maximum intensity values of the image before and after processing. The user can either save all the processed images or one selected image by clicking ‘Save all projections’ or ‘Save the current projection’. The saved data will be stored in the NPZ format, which is a compressed archive format used in Python’s *NumPy* library (Harris *et al.*, 2020 ▸) to store arrays and their corresponding metadata.

The third area is dedicated to displaying the images before and after processing and the plot of horizontal pixel intensities along the middle row. The display is updated automatically when sliding the cursor in the fourth area or when clicking on one item of the list in the second area. Finally, the fourth area allows editing of the displaying parameters. ‘Vmin_A’, ‘Vmax_A’, ‘Vmin_B’ and ‘Vmax_B’ define the data range of the image that the color map covers before and after processing, respectively.

### 2.1. Importing and handling data

*ProSPyX* requires a specific structure for naming the main folder and subfolders, and organizing data and metadata within the hierarchical data format (HDF) file. Usually, most of these requirements are satisfied when the data collected come from the SWING beamline at the SOLEIL synchrotron or the cSAXS beamline at the Swiss Light Source (SLS) of the Paul Scherrer Institut (PSI, Switzerland).

**Figure 7:**
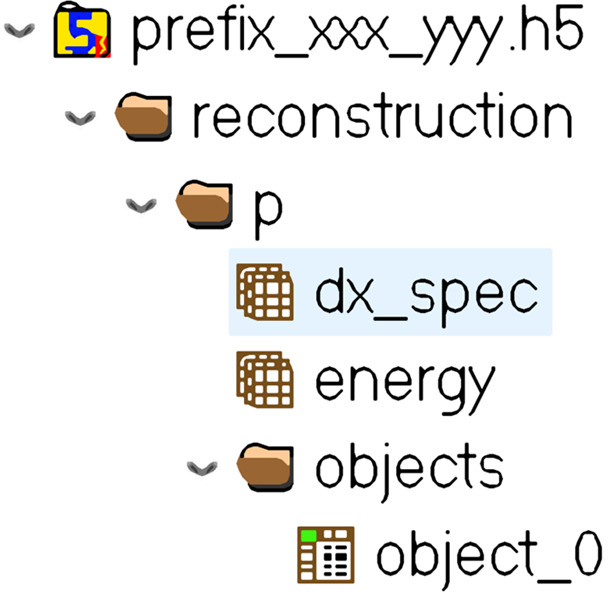
Minimal required architecture of the HDF file containing ptychographic reconstruction data. The pixel size and the energy are stored in ‘dx_spec’ and ‘energy’ inside the sub-group ‘p’ of the group ‘reconstruction’. The complex transmittivity function of the object is stored in ‘object_0’ inside the ‘objects’ group.

After the phase retrieval step, each ptychographic reconstruction should be stored in one HDF file with its metadata. Each file is saved separately in a subfolder. HDF files use a directory-like structure that allows the organization of the data within the file in many different structured ways (Koranne, 2011 ▸). For this software, the input data structure is illustrated in Fig. 7 ▸, with ‘object_0’, ‘dx_spec’ and ‘energy’ referring to the complex transmittivity function of the sample, pixel size and energy of acquisition, respectively. All subfolders must be stored in a unique main folder. The name of all HDF files and subfolders must contain the same prefix, which is unique to this dataset.

### 2.2. Data processing

#### 2.2.1. Experimental data acquisition

The dataset used to illustrate the data-processing procedure was acquired on the SWING beamline (Engblom *et al.*, 2019 ▸) at the French synchrotron SOLEIL. Spectral 2D X-ray ptychography was performed on a cone-shaped nickel metallic pillar with a mean diameter of 8 µm. A spectral 2D X-ray ptychography dataset was acquired by performing an energy scan from 8.3 keV to 8.4 keV with 1 eV steps, including the resonant energy of nickel, 8.333 keV. For each energy acquisition, the ptychographic scanning parameters were as follows: 242 diffraction patterns were recorded from a field of view of 14 µm × 12 µm with an exposure time of 100 ms per point. Considering the overhead, the total acquisition time for each 2D ptychographic image was 37 s. The ptychographic reconstruction was performed with the *PtychoShelves* software package (Wakonig *et al.*, 2020 ▸). A total of 200 iterations of the difference map (DM) algorithm (Thibault *et al.*, 2009 ▸), followed by 100 iterations of the maximum likelihood (ML) algorithm (Thibault & Guizar-Sicairos, 2012 ▸) were used for the ptychographic phase retrieval of each 2D image.

In order to allow the proper spectral analysis, we followed the pipeline of post-processing presented in Fig. 1 ▸ on the entire reconstructed images dataset. In the following sections, each of these steps and their implementation in the software will be described and illustrated using the acquired dataset.

#### 2.2.2. Constant and linear phase ramp-removal

The complex transmittivity function of the object, as any non-zero complex function, can be expressed in terms of its amplitude *A*(*x*, *y*) and phase φ(*x*, *y*). By combining these two terms, the expression of the complex transmittivity function of the object can be expressed in the following form,

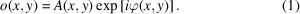

The reconstructed complex transmittivity function of the object after phase retrieval can additionally have constant and linear phase terms, namely

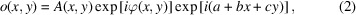

where *a*, *b* and *c* are real value coefficients. The constant phase term *a* arises from the fact that we cannot obtain absolute values of phase, which are recovered from the intensity values. Thus, an absolute value of the constant offset to the induced phase shift by the sample is uncertain in reconstruction since we can only obtain relative values. So, *a* is a bias to represent this uncertainty. The two linear phase terms *b* and *c* appear when the diffraction patterns are shifted relative to the center of the computational window of the Fourier space (Guizar-Sicairos *et al.*, 2011 ▸). They indicate the horizontal and vertical components of the phase gradient of the illuminated part of the object.

The algorithm proposed by Guizar-Sicairos *et al.* (2011 ▸) is implemented in this software to remove constant and linear phase-ramp terms. This algorithm relies on having an ROI outside the sample that contains only air, and hence, the phase shift is known to be zero for the calculation of the three real coefficients *a*, *b* and *c*. When the three coefficients are calculated, the phase ramp artifact is corrected by multiplying equation (2) by exp[−*i*(*a* + *bx* + *cy*)].

**Figure 8:**
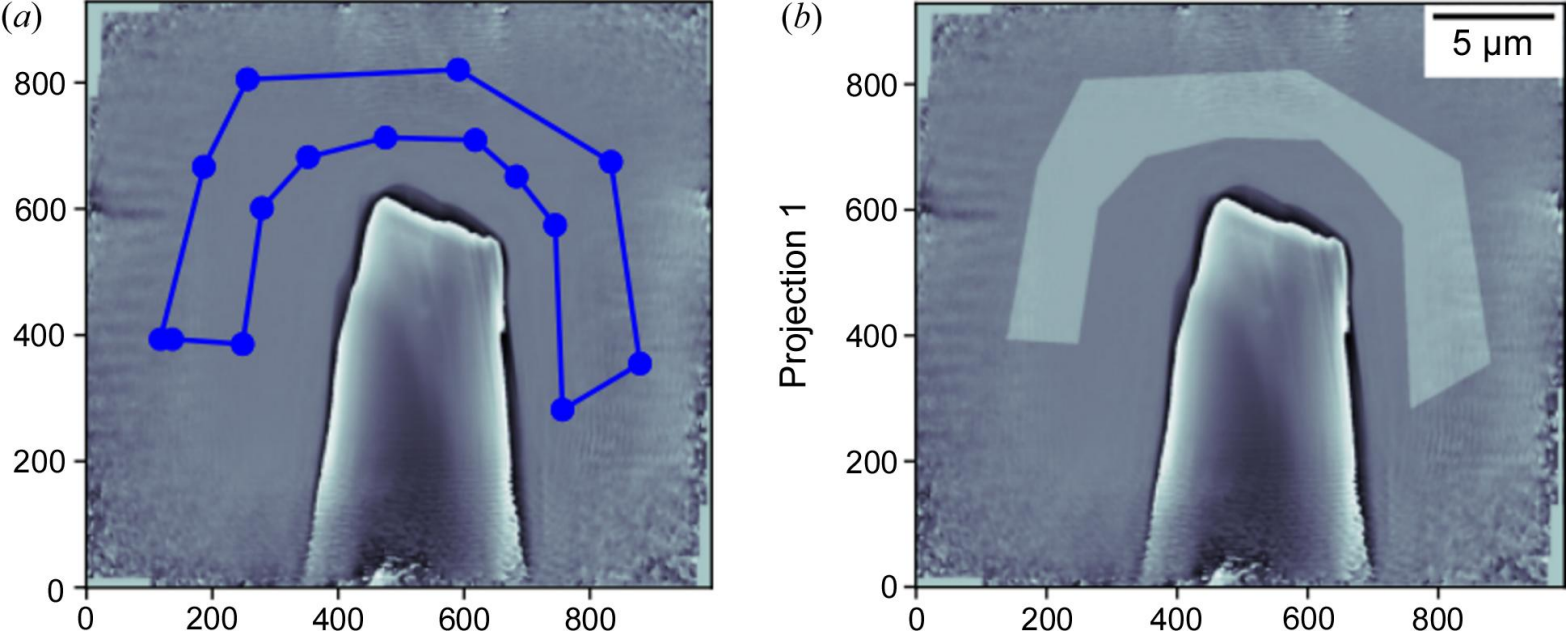
Definition of the air mask for the constant and phase ramp removal. (*a*) Drawing the air region manually using the mouse cursor. (*b*) Superimposition of the drawn mask with the current phase image.

The software provides the ‘Draw mask’ function to define the ROI outside the sample. The ‘Draw mask’ push button triggers the apparition of a new window where it is possible to draw the ROI outside the sample (Fig. 8 ▸), where the phase signal should be zero using the cursor. ‘Add mask’ or ‘Mask all’ buttons allow the user to visually superimpose the ROI with the currently selected phase image or the entire set of phase images. The result of the overlying is seen directly in area 3 [see Fig. 8 ▸(*b*)]. In the case where the user is not satisfied with the ROI drawn, they can first select ‘Remove mask’ or ‘Remove all mask’ to clear the visual superposition and then draw a new mask using the ‘Draw mask’ function.

When the ‘Apply mask’ button is pressed, the chosen ROI and the currently selected phase image are taken as input to the constant and linear phase ramp-removal algorithm. If ‘Apply all mask’ is pressed, the entire dataset is processed with the same chosen ROI.

#### 2.2.3. Phase unwrapping

The phase contrast image is calculated from the complex transmittivity of the sample using the arc-tangent function. The phase values are constrained to the range ±180° due to the arc-tangent limits. Thus, the phase is calculated modulo 2π and does not necessarily reflect the absolute value of the measured phase signal; this is called the wrapped phase problem. Phase unwrapping is the process of recovering unwrapped phase values from the wrapped phase map. This is done by identifying discontinuities in the 2D map wrapped phase and adding or subtracting 2π to the phase values at these locations to restore its original range.

2D phase unwrapping is the second step in the data-processing pipeline. The phase unwrapping algorithm used here is described by Herráez *et al.* (2012 ▸), which is the same as that implemented in the *scikit-image* package (Pedregosa *et al.*, 2011 ▸).

The ‘Unwrap’ button applies phase unwrapping on the currently selected image. ‘Unwrap all’ applies the phase unwrapping on the total loaded phase images (see Fig. 1 ▸).

#### 2.2.4. Normalization

**Figure 9:**
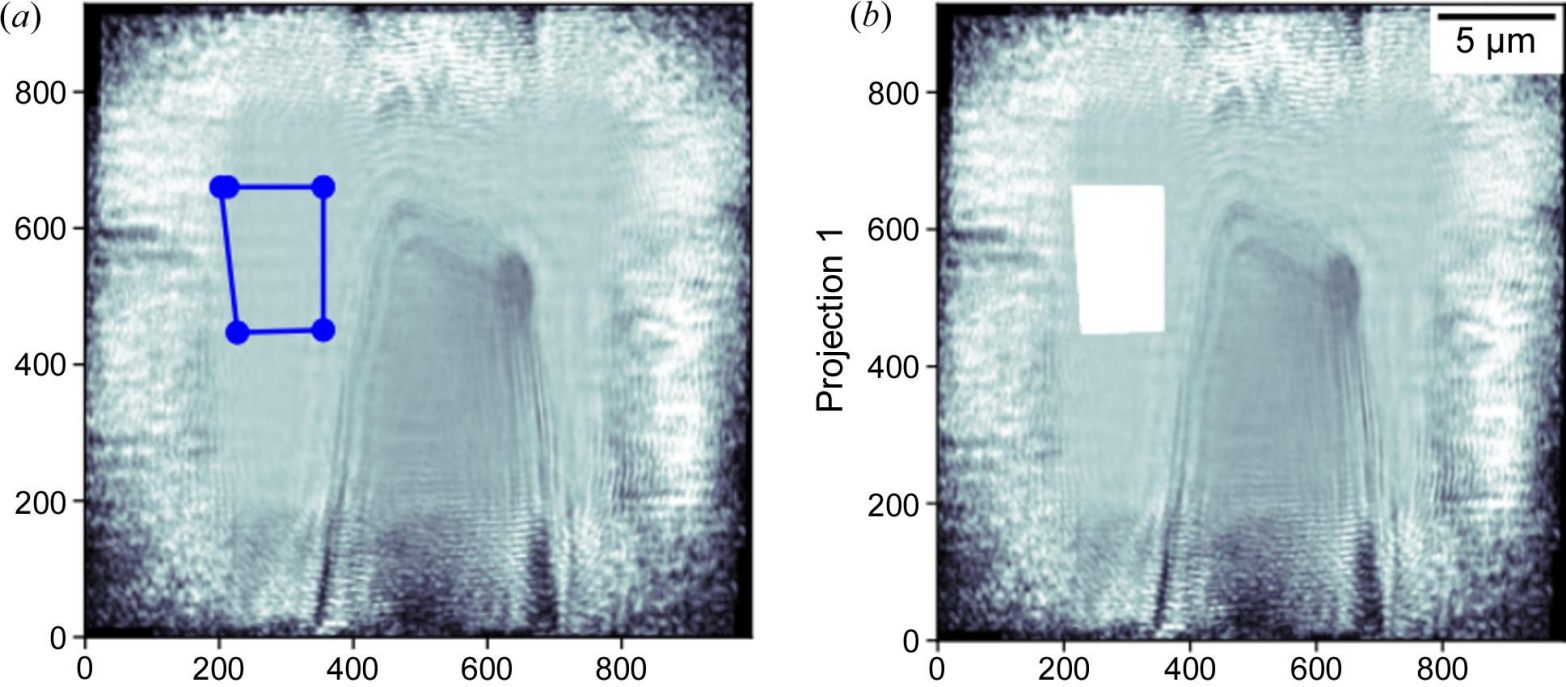
Definition of the air region for the normalization of the amplitude images. (*a*) Drawing the air region manually using the mouse cursor. (*b*) Superimposition of the drawn mask with the current amplitude image.

Normalization involves dividing the amplitude image by the mean amplitude in the air region. As with the constant and linear phase ramp-removal described above, the air region is defined using the ‘Draw mask’ button followed by ‘Add mask’ or ‘Mask all’ for the superimposition with an amplitude image or the entire set of amplitude images selected (Fig. 9 ▸). Finally, by pushing ‘Normalization’ or ‘Normalization all’ [see Fig. 3 ▸(*b*)] the normalization of either one selected amplitude image or all the amplitude images in a dataset is performed.

#### 2.2.5. Conversion to the product of the sample linear attenuation coefficient and thickness

To determine the spectra extracted from the absorption contrast images later on, it is necessary to consider the linear attenuation coefficient μ and thickness of the sample. X-ray attenuation decreases exponentially with the thickness of the material traversed. X-ray intensity is the squared amplitude of the complex transmissivity function of the sample. Using the Beer–Lambert law, the equation to calculate the image of the product between the linear attenuation coefficient and thickness of the sample is

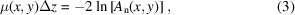

where *A*_n_(*x*, *y*) is the image of the amplitude of the complex transmittivity function normalized by the mean amplitude of a region outside the sample. The buttons labeled ‘Convert to (μ*sample thickness)’ and ‘Convert to (μ*sample thickness) all’ [see Fig. 3 ▸(*b*)] have been created for this specific task, for one image from the absorption dataset or from the entire dataset.

#### 2.2.6. Pixel interpolation

Spectral 2D X-ray ptychography, like any other spectral imaging approach, consists of performing an energy scan within a specific energy range. In ptychography, the pixel size in each reconstructed phase or absorption contrast image is related directly to the energy, which is inversely proportional to the wavelength of the incident photons,

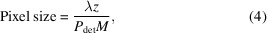

where *z* is the distance between the sample and detector, λ the wavelength of the incident X-ray (inversely proportional to photon energy), *P*_det_ represents the pixel size of the detector and *M* is the number of columns or rows of the detector array. As one can see, the pixel size decreases with increasing energy. Consequently, the image size is different from one acquisition to another, making it necessary to rescale all the images to the same pixel size. One approach is to interpolate the pixels of all images with respect to the image with the highest energy since it has the smallest pixel size. All interpolated images are rescaled with the scaling factor *f*,

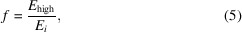

where *E*_high_ is the highest energy of acquisition and *E*_*i*_ is the acquisition energy of the image to be interpolated. The new pixel size of the interpolated image is equal to

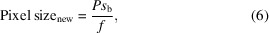

where *Ps*_b_ is the pixel size before interpolation. All absorption and phase shift images are interpolated by clicking ‘Interpolate’. The script will first fetch the image with the highest energy and then start the spline interpolation step. As mentioned before, the pixel size and energy information must be written in the HDF file following the file architecture discussed above; otherwise, the interpolation will not be performed.

#### 2.2.7. Alignment

For any spectral imaging dataset, the sample image must be located at the same coordinates in the obtained images for further processing or information extraction. Nevertheless, during the experiment, the sample can shift by arbitrary distances from the initial location due to instrument limitations or other technical issues. The mis­alignment can be numerically adjusted afterwards using alignment algorithms. In the context of far-field ptychography with hard X-rays, phase contrast offers a higher signal-to-noise ratio than absorption contrast, which makes sense to use the phase contrast images for an accurate shift estimation. The button ‘Align all’ on the software allows the registration of the entire dataset relative to the reference image. Here, the reference is the phase image with the highest energy for the phase shift contrast images. The algorithm used in this software to determine the shifts with pixel or subpixel accuracy is phase cross-correlation (Guizar-Sicairos *et al.*, 2008 ▸) from the *scikit-image* package. The moving image is shifted using spline interpolation of cubic order by employing the shift function in the *SciPy* library (Virtanen *et al.*, 2020 ▸). Once all the phase shift images are correctly aligned, the user should switch to absorption contrast images and press the ‘Align all’ button again. However, in this case, the software does not estimate new shifts; instead, it uses the previously estimated shifts from the phase contrast images to correct any misalignment in the absorption contrast images. Note that if the user chooses to align the absorption images first, a pop-up error message will appear instructing them to calculate the shifts from the phase contrast images.

### 2.3. Spectra extraction of the refractive index decrement and absorption index

Once the post-processing discussed in Section 2.2 is completed, it is now possible to convert the images of two contrasts into quantitative maps of the refractive index decrement δ(*x*, *y*) and absorption index β(*x*, *y*), with β and δ being the imaginary and the real part of the complex refractive index *n*,

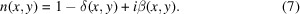

The complex refractive index *n* is also related to atomic scattering factors *f*_0_, *f*′ and *f*′′ by

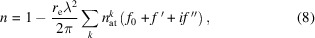

where *r*_e_ is the classical electron radius, λ the wavelength of the incident X-rays and 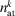 is the atomic density of the *k*th element in the sample. *f*_0_ represents coherent scattering, *f*′ reflects the dispersive nature of X-ray scattering due to the energy dependence of the scattering process and *f*′′ represents the X-ray absorption by the atom which leads to a decrease in the intensity of the transmitted X-rays. The terms *f*_0_ and *f*′ belong to the real part of the complex refractive index *n*, which means they are related to the refractive index decrement δ. The absorption index β is related to *f*′′.

By clicking ‘Contrast conversion’, all the uploaded phase images on the software are converted for each image as follows,

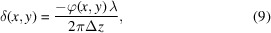

where φ(*x*, *y*) is the input phase image and Δ*z* is the sample thickness defined by the user.

The absorption contrast images are calculated according to equation (10),

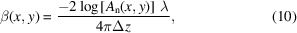

where *A*_n_(*x*, *y*) refers to the normalized amplitude images of the complex transmittivity function of the sample.

In cases where the thickness of the sample is unknown, *ProSPyX* offers a practical solution. It enables the conversion of phase contrast images to the product between the refractive index decrement δ(*x*, *y*) and the thickness of the sample Δ*z*, and images related to the absorption into the product between the absorption index β(*x*, *y*) and thickness of the sample Δ*z*. By clicking ‘Contrast conversion’, a pop-up dialog window appears, prompting the user with the question ‘Do you know the thickness of your sample?’ The user can choose either yes or no.

**Figure 10:**
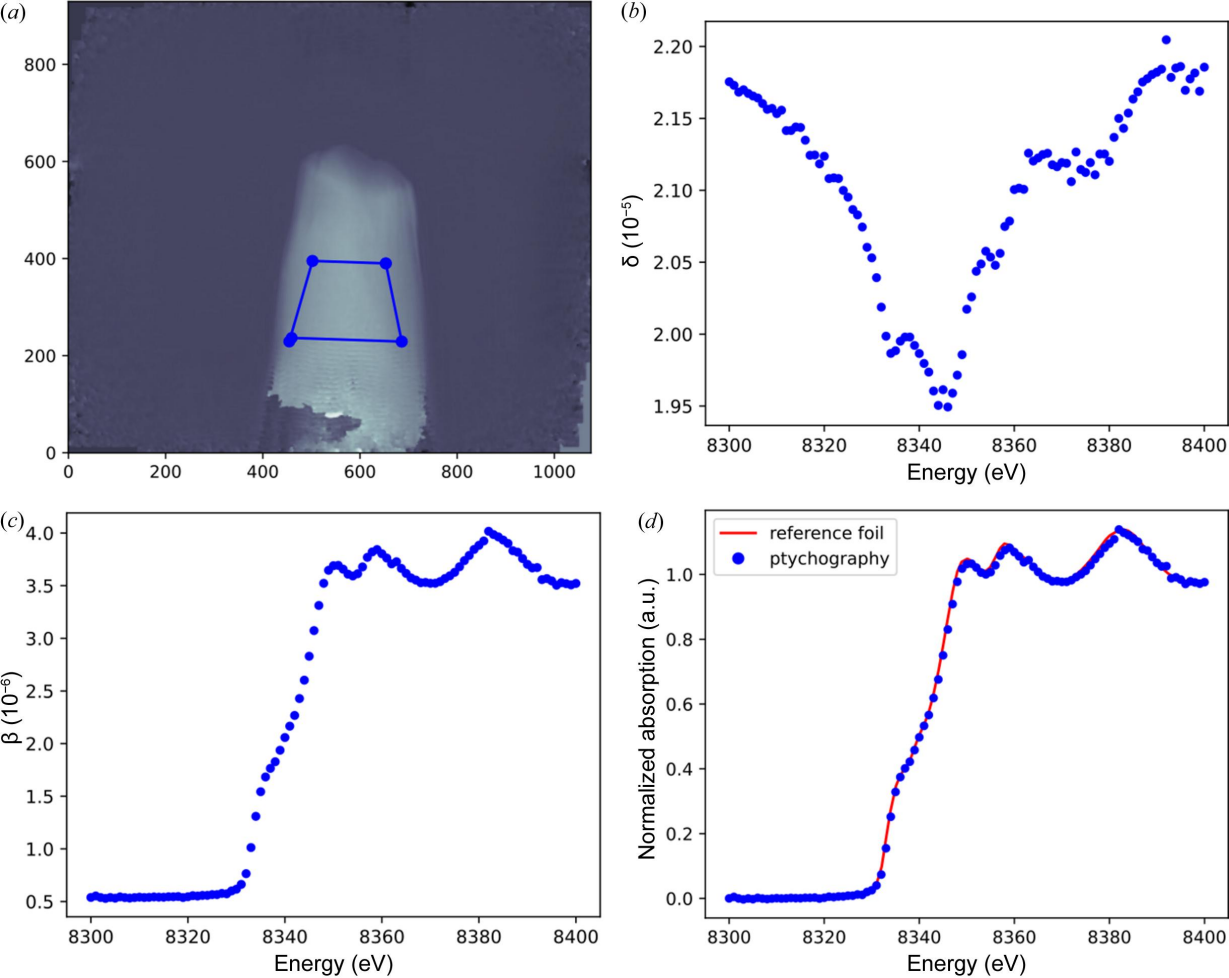
Spectra extraction of the refractive index decrement and absorption index. (*a*) Definition of the ROI using the mouse cursor to extract spectra. (*b*) Calculated spectrum related to phase shift within the defined ROI. (*c*) Calculated spectrum related to absorption within the defined ROI. (*d*) Comparison between two normalized absorption spectra of nickel. The blue and red normalized spectra are obtained using X-ray spectral ptychography and conventional X-ray spectroscopy, respectively.

The spectra related to phase shift and absorption are extracted from one ROI inside the δ(*x*, *y*) and β(*x*, *y*) contrast-based images. Manually, this can be done by averaging the δ or β values inside the defined ROI for each image acquired using the known energy of incident photons. This approach is implemented in our software. First, the user must click ‘Draw ROI’. This triggers the appearance of the new window where it is possible to define the desired ROI using the cursor. Next, by clicking ‘Mean_ROI’, the mean inside each ROI of the δ(*x*, *y*) and β(*x*, *y*) images dataset will be calculated automatically. The new figure will display the two spectra (see Fig. 10 ▸). These spectra are saved in the same path where the application is executed for further analysis with other spectroscopy software.

The extracted spectra can help with the chemical speciation of the sample. For example, it can serve to determine the exact value of the energy of the absorption edge. At the absorption energy, the phase shift values should be the smallest compared with other values because the incident photons are absorbed at this level. Moreover, these spectra give the user crucial spectral information about the sample, such as the valence or chemical nature of the probed element. It allows for extraction of important spectroscopic information on the chemical evolution of the probed element. In this particular case, we find the spectrum of the pure metal.

To evaluate the accuracy of the method, the absorption spectrum obtained here for 7.5 µm sample thickness was compared with a second absorption spectrum acquired from nickel foil with conventional X-ray absorption spectroscopy on beamline BM30 at ESRF (SSHADE/FAME, 2017 ▸; Proux, 2018 ▸). This spectrum was taken within the energy range [8102.0, 9303.0] eV with a resolution of 0.42 eV. After normalization with the *Larch* software (Newville, 2013 ▸), where the normalization process involved using a pre-edge region of [−45, −30] eV for both spectra and a post-edge region of [44.85, 54.85] eV for the absorption spectrum of X-ray spectral ptychography and [45, 55] eV for the nickel foil acquired with conventional X-ray spectroscopy, a constant polynomial function was used to achieve normalization; both the pre-edge and the post-edge region values, as well as the polynomial constant, were set to default by *Larch* after importing both spectra. The comparison was conclusive about the accuracy of X-ray spectral ptychography. Qualitatively [see Fig. 10 ▸(*d*)], the comparison between the two absorption spectra shows a good agreement. Quantitatively, the normalized root mean squared error between the two absorption spectra was determined to be 6%.

## 3. Conclusions

This paper introduced *ProSPyX*, an open-source Python software developed with *PyQt5*. This software is specifically designed to process large X-ray spectral ptychography datasets containing phase and absorption contrast images. *ProSPyX* provides a friendly GUI with access to various analysis tools. These tools include linear phase ramp removal, phase unwrapping, amplitude image normalization, and conversion of the sample linear attenuation coefficient and thickness. All of these operations can be performed interactively and directly within the software. *ProSPyX* performs pixel interpolation on both absorption and phase shift datasets simultaneously. During the alignment process, the shifts estimated from the phase contrast images to correct the mis­alignment are applied on both contrasts since the signal-to-noise ratio in phase contrast is higher. Once the post-processing is complete, contrast conversion can be carried out to extract spectra related to phase shift and absorption. *ProSPyX* also allows users to save the results of each processing step and isolate an ROI using the crop tab.

Currently, *ProSPyX* allows users to save extracted spectra for later examination using the X-ray spectroscopy software to identify resonant chemical species in specific areas of interest in the sample. However, as a future development, we plan to expand *ProSPyX* into full software for XANES ptychography by embedding processing and analysis functionalities, which can be found in established reference software for spectroscopy data. The added functionalities will offer more practicality to users, as they will have access to all the necessary tools within a single software package, eliminating the need to run multiple software tools simultaneously. Our vision is to introduce distinct tabs within *ProSPyX*. These include a data importation tab; a XANES analysis tab where the user can extract essential information about the oxidation state, coordination environment and bonding of elements within the sample; a data fitting tab that enables the user to perform curve fitting to derive quantitative information from XAS spectra; and a spectral simulation tab where the users will have the capability to simulate X-ray absorption spectra based on theoretical models. This aids in the interpretation of experimental results and refines structural information. Finally, with the increasing availability of computational resources, it may be convenient to introduce a machine-learning tab that enables users to extract hidden information and patterns from XAS data, assisting in data analysis and interpretation. By incorporating these functionalities into *ProSPyX*, we aim to provide a comprehensive and user-friendly software platform for XANES ptychography, meeting the diverse needs of researchers and eliminating the need for multiple external software tools.

## 4. Software and data availability

The source code and the latest developments of *ProSPyX* are available on GitHub (https://github.com/RedhouaneBJM/ProSPyX). The data used to demonstrate the software functionality are open source and available at https://10.5281/zenodo.10000775.
